# The Kinetic Changes of Systemic Inflammatory Factors during Bevacizumab Treatment and Its Prognostic Role in Advanced Non-small Cell Lung Cancer Patients

**DOI:** 10.7150/jca.30478

**Published:** 2019-08-28

**Authors:** Butuo Li, Shijiang Wang, Cheng Li, Meiying Guo, Yiyue Xu, Xindong Sun, Jinming Yu, Linlin Wang

**Affiliations:** 1 Tianjin Medical University Cancer Institute and Hospital, National Clinical Research Center for Cancer, Key Laboratory of Cancer Prevention and Therapy, Tianjin, China; 2 Department of Radiation Oncology, Tianjin Medical University, Tianjin, China; 3 Department of Radiation Oncology, Shandong Cancer Hospital and Institute Affiliated to Shandong University, Shandong First Medical University and Shandong Academy of Medical Science, Jinan, China; 4 Department of Dean's Office, Shandong Cancer Hospital and Institute Affiliated to Shandong University, Shandong First Medical University and Shandong Academy of Medical Science, Jinan, China; 5 Department of Radiation Oncology, School of Medicine, Shandong University, Jinan, China

**Keywords:** systemic immune status, mixed effect model, longitudinal changes, prognostic factors, survival analysis

## Abstract

**Background**: Bevacizumab combined with chemotherapy is still one of the standard options for treatment of advanced non-small cell lung cancer (NSCLC) patients without driver mutations. Serum inflammatory factors, representing the systemic immune status, are shown to have complicated relationships with tumor angiogenesis, and proved to be associated with survival of advanced NSCLC patients. However, the information from the baseline factors is relatively limited, which cannot reflect the dynamic changes of systemic immune status during bevacizumab treatment. We, thus, attempted to evaluate longitudinal kinetics of systemic inflammatory factors during treatment of bevacizumab and to explore their predictive role in treatment response and patient outcomes in advanced NSCLC.

**Method**: Systemic inflammatory factors (neutrophil/lymphocyte (NLR), platelet/lymphocyte (PLR), neutrophil×platelet/lymphocyte (SII) and lymphocyte/monocyte (LMR)) and clinical variables were collected and analyzed from 161 advanced NSCLC patients treated with bevacizumab. Mixed effect regression models were first performed for longitudinal analysis of the changes of serum inflammatory factors during bevacizumab treatment. Then, univariate and multivariate Cox models were used for overall survival (OS) and progression free survival (PFS) analyses to determine the independent prognostic factors.

**Results**: In the first 6 cycles of bevacizumab treatment, patients with complete response/partial response (CR/PR) had a -0.11, -0.066, -0.15, and 0.073 change every 2 cycles in transformed NLR (95%CI: -0.19--0.03, p=0.008), PLR (95%CI: -0.12--0.013, p=0.015), SII (95%CI: -0.23--0.05, p<0.001) and LMR (95%CI: 0.049-0.14, p=0.036), respectively, compared to patients with progressive disease (PD). With respect to analysis of the longitudinal changes before progression, patients experienced a significant increase in transformed NLR (Coef=0.09, 95%CI: 0.019-0.17, p=0.014), PLR (Coef=0.05, 95%CI: 0.002-0.10, p=0.04), and SII (Coef=0.091, 95%CI: 0.015-0.17, p=0.019), but a decrease in transformed LMR (Coef=-0.08, 95%CI: -0.14-0.018, p=0.012). On multivariate Cox model analyses, decrease of LMR (HR=0.62, 95% CI: 0.4-0.96, p=0.033) was shown to be the independent risk factor for PFS; and low level of baseline LMR (HR=0.4, 95% CI: 0.17-0.94, p=0.036), increase of NLR (HR=2.36, 95%CI: 1.25-4.44, p=0.008), and decrease of LMR (HR=0.42, 95%CI: 0.18-0.97, p=0.041) were the independent risk factors for death.

**Conclusion**: The activation of systemic immune status evaluated by the kinetic changes of serum inflammatory factors was associated with good response to bevacizumab; however, the suppressive status may indicate the resistance to bevacizumab. Dynamic changes of systemic inflammatory factors also had prognostic value in predicting outcomes of advanced NSCLC patients treated with bevacizumab.

## Introduction

In the new era of target therapy, bevacizumab combined with chemotherapy is still one of the standard options for patients with advanced non-small cell lung cancer (NSCLC), especially for those without driver mutations.^1^-^3^ However, the clinical benefit from bevacizumab for advanced NSCLC patients is disappointing, with the median overall survival (OS) of patients ranging from 12.3 months to 24.3 months.^1^-^3^ Recently, the combination of bevacizumab and immune-checkpoint inhibitors has been investigated and shown to have promising outcomes in the treatment of advanced NSCLC.^4^-^6^ Nevertheless, the time point of immunotherapy administration, and the reliable biomarkers for identifying suitable patients are largely unknown. Thus, the investigation of dynamic changes of immune status during treatment of bevacizumab may not only help to screen patients benefiting from bevacizumab, but also provide clinical evidence for combination of bevacizumab and immunotherapy.

More and more evidences showed that there is a complicated relationship between immune activities and angiogenesis in cancer.^7^-^9^ On the one hand, antiangiogenic therapy with bevacizumab (VEGF blockage) was found to increase infiltration of T lymphocyte into tumors, inhibit proliferation of T-regulatory cell, and enhance maturation of dendritic cells, which was demonstrated to have positive roles in stimulation of immunological response.^10^, ^11^ On the other hand, the tumor response of antiangiogenic therapy could also be influenced by immune cells.^12^, ^13^ Bone marrow-derived immune cells could be attracted to the tumor tissue by different stimuli or cytokines, 14 and induce a VEGF-independent angiogenesis, rendering the resistance to bevacizumab.^15^ Interestingly, serum inflammatory cells representing the systemic immune status, could not only reflect the status of tumor microenvironment; 16 but also play an important role in promoting proliferation of endothelial cells, inducing neovascularization and re-growth of tumor during bevacizumab treatment.^17^-^20^ Thus, we propose that the systemic immune cells may be one of the indirect markers for monitoring the clinical response of bevacizumab in advanced NSCLC.

Systemic inflammatory factors, including neutrophil to lymphocyte ratio (NLR) 21, platelet to lymphocyte ratio (PLR)^22^, systemic immune inflammation index (SII)^23^, lymphocyte to monocyte ratio (LMR)^24^, lactate dehydrogenase (LDH)^25^, and C-Reactive Protein (CRP)^26^ and others, have been reported to be useful for predicting patient outcomes in various malignant solid tumors. In advanced NSCLC^23^ and colorectal cancer 27, high baseline NLR was further found to be associated with poor survival of patients receiving bevacizumab treatment. However, the immune status of both system and tumor microenvironments was not static, but with dynamic changes during bevacizumab treatment. Therefore, the study on the longitudinal changes of systemic inflammatory factors during bevacizumab treatment and their potential relation with the efficacy of bevacizumab may be more informative, and will be more precise for selecting appropriate patients to receive clinical benefits.

Consequently, the purpose of our study was to evaluate longitudinal kinetics of systemic inflammatory factors during bevacizumab treatment in advanced NSCLC patients and to explore their predictive role in the treatment response and patient outcomes.

## Materials and Method

### Patient population

This study was carried out in accordance with the principles of the 1975 Declaration of Helsinki and its later amendments or comparable ethical standards, and was approved by the Ethics Committee of Shandong Cancer Hospital Affiliated to Shandong University (No. SDTHEC201806002). For this retrospective study, formal consent was not required, while all data were kept confidential. 161 consecutive patients with advanced (IIIB/IV stage) NSCLC who underwent bevacizumab plus standard chemotherapy in Shandong Cancer Hospital, between June 2011 and February 2018, were included in this study.

The medical records of each patient were reviewed with respect to consecutive laboratory complete blood count during bevacizumab treatment and other clinical factors, including gender, age, smoking status, EGFR status, anatomical location (central or peripheral), and the presence or absence of liver, brain, or bone metastases. To ensure consistent data collection, all the data were collected retrospectively by using uniform database templates. Tumor response was assessed according to Response Evaluation Criteria in Solid Tumors (RECIST) version 1.1. Progression free survival (PFS) and overall survival (OS) was defined as the time from the initiation of bevacizumab to the date of progression and the date of death or last follow-up, respectively.

Inflammatory factors included NLR, PLR, SII, and LMR. The NLR data were defined as the ratio of absolute neutrophil count to absolute lymphocyte count; PLR data, the ratio of absolute platelet count to lymphocyte count; SII data, calculated as platelet count × neutrophil count/lymphocyte count; and LMR data, constructed with the ratio of the absolute lymphocyte count to absolute monocyte count. The changes were defined as differences between inflammatory factors before treatment and inflammatory factors after 6 cycles of bevacizumab with chemotherapy.

### Statistical analysis

To assess the dynamic changes of inflammatory factors during bevacizumab treatment, two analyses were performed in this study. The first analysis investigated the longitudinal assessment of inflammatory factors from the initiation of bevacizumab to the sixth cycle of treatment. Whereas, the second analyzed the longitudinal assessment of serum inflammatory factors during four cycles before progression (in patients with progression) or last follow-up (in patients with no progression) during bevacizumab treatment. Mixed effect regression models with per-patient random intercept and slope were performed for the longitudinal analyses of serum inflammatory factors. The value of serum inflammatory factors was then transformed to be normally distributed to perform a better regression model using a zero-skewness log transformation in STATA v12.0. Transformed NLR, PLR, SII and LMR was defined as ln(NLR-0.18), ln(PLR + 15.9), ln(SII-14.7), and ln(LMR-0.17) in first analysis, and ln(NLR-0.20), ln(PLR+6.9), ln(SII+19.9), and ln(LMR-0.055), respectively, in second analysis.

All patients were dichotomized into “low” and “high” groups according to the inflammatory factors at basement. The cut-off values of baseline NLR, PLR, SII, and LMR were defined as 3.87, 156, 824, and 2.37, respectively, according to receiver operating characteristics (ROC) analysis. Univariate and multivariate Cox models were applied in OS and PFS analyses to assess the independent prognostic values among clinical factors and serum inflammatory factors. Variables with p<0.1 in univariate analyses were included in multivariate Cox models. Results were presented as hazard ratios (HR) with 95% confidence intervals (95% CIs). All p-values were two-sided, with statistical significance defined as p<0.05. Survival analyses and mixed effect models were conducted using SPSS 24 and figures were carried out using GraphPad Prism 7.

## Results

### Patient Characteristics

A total of 161 advanced non-squamous NSCLC patients treated with bevacizumab were enrolled in our study; and 74 patients were dead at the end of follow-up. Baseline characteristics are shown in Table 1.

### Longitudinal analysis of systemic inflammatory factors during first 6 cycles of bevacizumab treatment

A mixed effect regression analysis with per-patient random intercept was first used to analyze systemic inflammatory factor changes during the first 6 cycles of bevacizumab treatment. All patients were divided into complete response/partial response (CR/PR), stable disease (SD), and progressive disease (PD) groups, according to the response to bevacizumab treatment.

**Figure 1:**
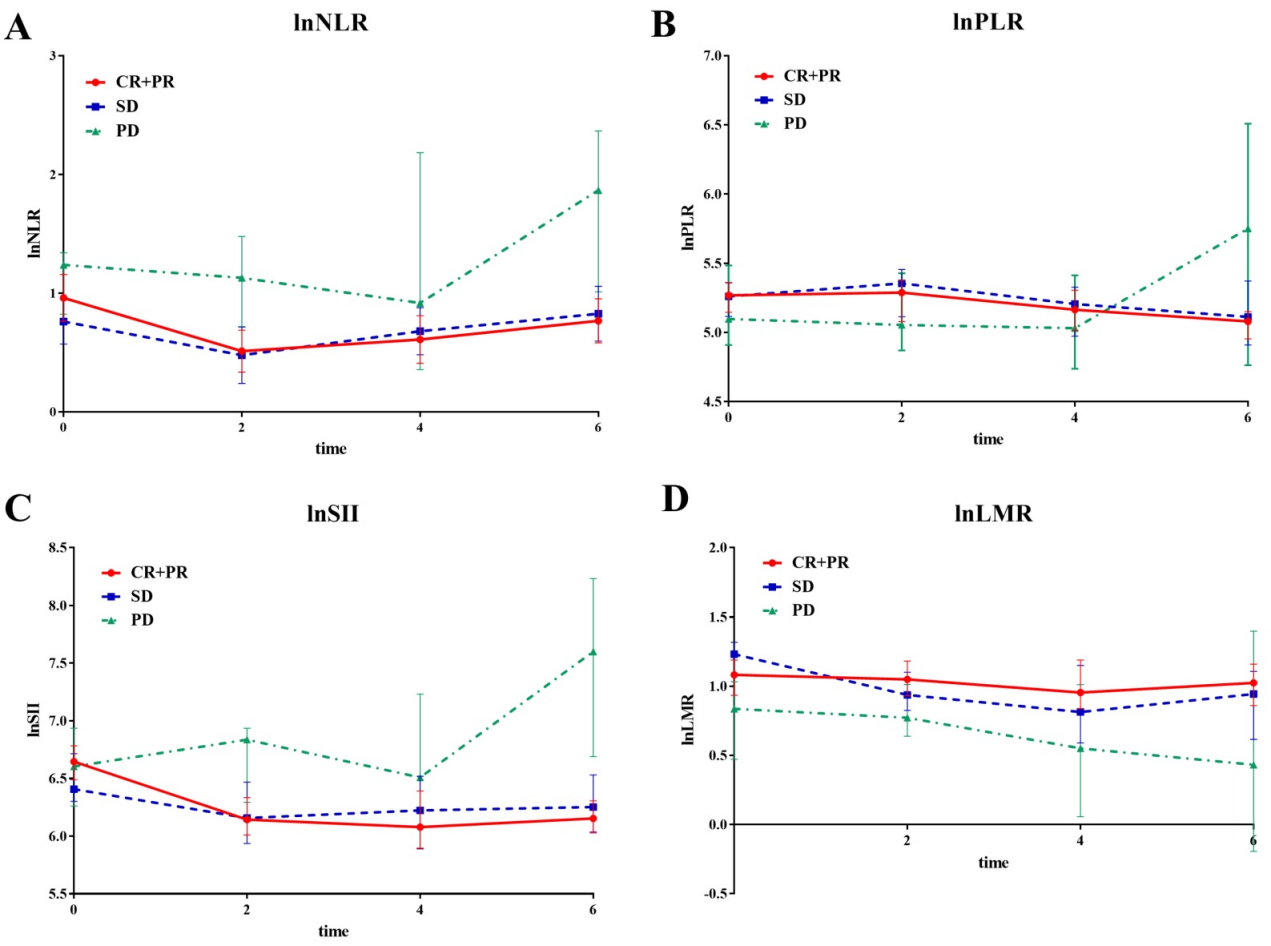
The line chart of median value of systemic inflammatory factors divided by response status during first 6 cycles of bevacizumab. The line chart of median value of systemic inflammatory factors at baseline, 2nd, 4th and 6th cycle treatment divided by tumor response (CR/PR, SD and PD) during first 6 cycles of bevacizumab. **A** lnNLR; **B** lnPLR; **C** lnSII; **D** lnLMR.

Patients with response of CR/PR had -0.11, -0.066, -0.15, and 0.073 changes in transformed NLR (95%CI: -0.19--0.03, p=0.008), PLR (95%CI: -0.12--0.013, p=0.015), SII (95%CI: -0.23--0.05, p <0.001), and LMR (95%CI: 0.049-0.14, p=0.036), respectively, every 2 cycles of bevacizumab treatment, compared to patients with PD. There was also a statistical significance in the decline of transformed SII in patients with SD (Coef=-0.098, 95%CI: -0.18--0.016, p=0.019), when compared with patients with PD. However, there was no statistical significance in changes of transformed NLR, PLR and LMR between patients with SD and PD. Notably, when combining patients with SD and PD, patients with CR/PR experienced significant changes, when compared with SD/PD patients on transformed NLR (Coef=-0.05, 95%CI: -0.10-0, p=0.048), SII (Coef=-0.065, 95%CI: -0.11--0.018, p=0.007), and LMR (Coef=0.057, 95%CI: 0.016-0.098, p=0.007), respectively, but not on transformed PLR (Coef=-0.027, 95%CI: -0.060--0.01, p=0.1) (Figure 1).

### Longitudinal analysis of systemic inflammatory factors before progression of bevacizumab treatment

All enrolled patients were divided into 'progression' and 'no progression' groups depending on the disease evaluation at the end of follow-up. The mixed effect regression analysis was then performed to compare the last 4 cycle changes in systemic inflammatory factors between both groups.

**Figure 2:**
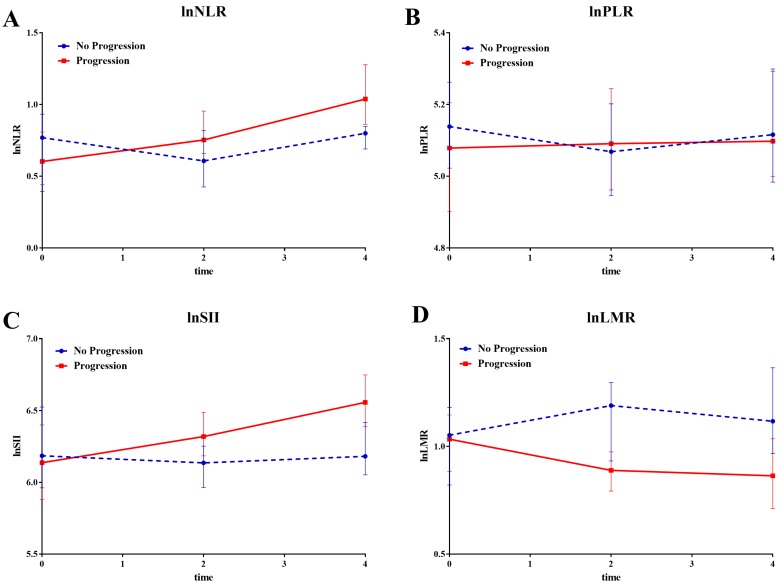
The line chart of median value of systemic inflammatory factors divided by disease status at last follow-up. The line chart of median value of systemic inflammatory factors divided by disease status (Progression and No Progression) at last follow-up during the last four cycles. **A** lnNLR; **B** lnPLR; **C** lnSII; **D** lnLMR.

Compared to patients in no progression group, patients experienced significant increase in transformed NLR (Coef=0.09, 95%CI: 0.019-0.17, p=0.014), PLR (Coef=0.05, 95%CI: 0.002-0.10, p=0.04), and SII (Coef=0.091, 95%CI: 0.015-0.17, p=0.019) in progression group, but a decrease in transformed LMR (Coef=-0.08, 95%CI: -0.14-0.018, p=0.012) (Figure 2).

### Survival analysis

With a median follow-up of 16.6 months (2.3-86.3 months), the median PFS and OS of the whole group were 8.1 months (95% CI: 5.8-10.4) and 27.0 months (95%CI: 20.8-34.9), respectively. The effect of potential prognostic factors for PFS and OS were analyzed using Cox models (Table 2 and Table 3).

On univariate analyses, smoking history, anatomical location with the central tumor, and bone and liver metastases were found to be predictive for inferior OS and PFS. In the field of systemic inflammatory factors, high baseline NLR and SII, low baseline LMR, increased NLR and SII, and decreased LMR after 6 cycles of bevacizumab were significantly associated with worse PFS. Whereas patients with high baseline NLR, low baseline LMR, increased NLR and SII, and decreased LMR after 6 cycles of bevacizumab were all significantly related to the high risk of death.

On multivariate analyses, anatomical location of the central tumor, liver metastasis and decreased LMR (HR=0.62, 95% CI 0.4-0.96, p=0.033) remained independent risk factors for PFS. As for OS, liver metastasis, low baseline LMR (HR=0.4, 95% CI 0.17-0.94, p=0.036), increased NLR (HR=2.36, 95%CI 1.25-4.44, p=0.008), and decreased LMR (HR=0.42, 95%CI 0.18-0.97, p = 0.041) were the independent risk factors for death (Table 2 and Table 3).

**Figure 3:**
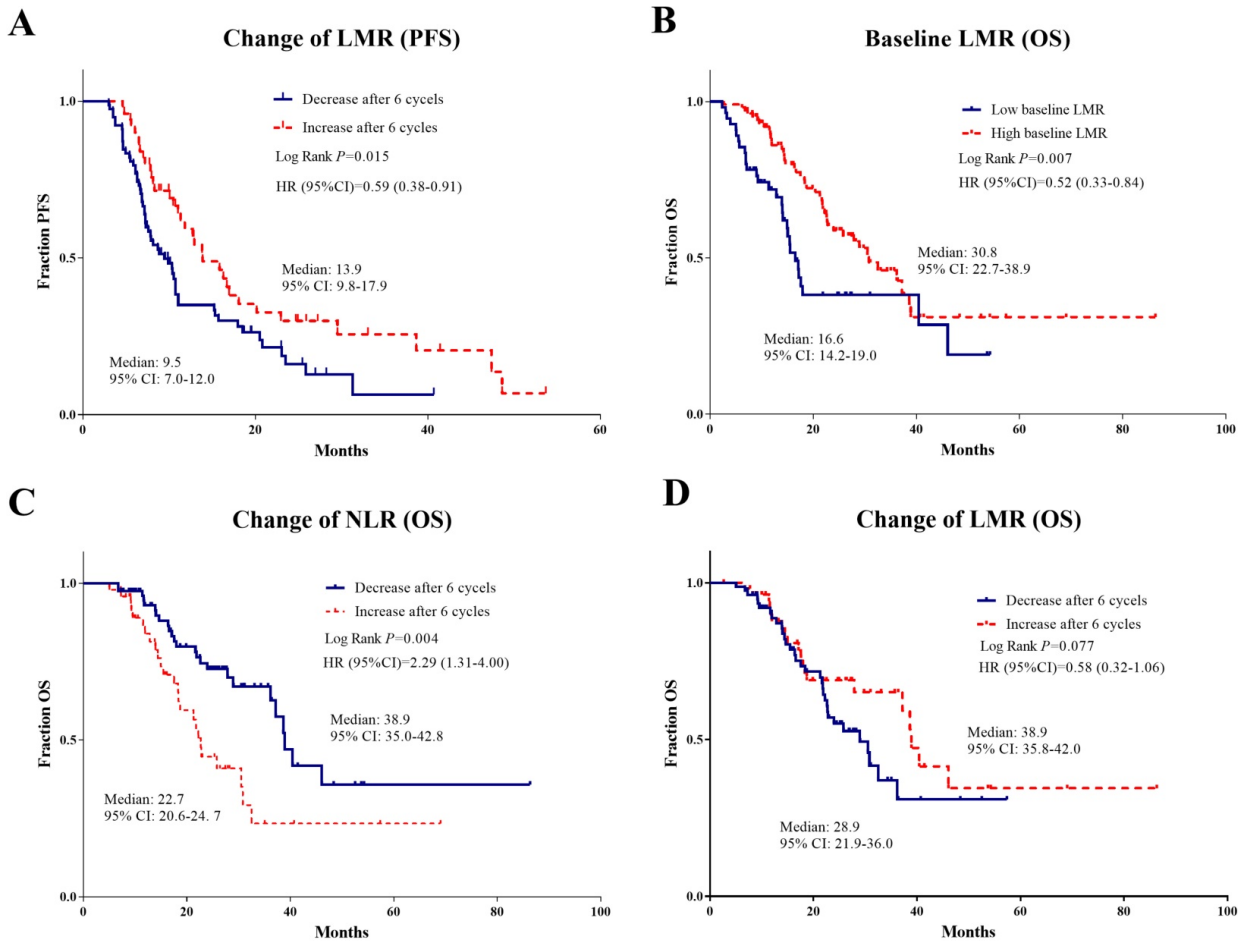
Kaplan Meier survival plots of independent risk factors for PFS and OS with respect to systemic inflammatory factors. Kaplan Meier survival plots of independent risk factors for PFS (**A**) and OS (**B, C, D**) with respect to systemic inflammatory factors in advanced NSCLC patients treated with bevacizumab: **A** Change of LMR for PFS; **B** Baseline LMR for OS; **C** Change of NLR for OS; **D** Change of LMR for OS.

The independent risk inflammatory factors for PFS and OS were then further confirmed using Kaplan-Meier analyses, which showed the good capacity in prognostic prediction (Figure 3).

## Discussion

Antiangiogenetic therapy with bevacizumab has been proposed to play a critical role in the alleviation of immunosuppression in tumor microenvironments. 10, 11, 15 Serum inflammatory factors, representing the systemic immune status, were found to be associated with the density of immune cells in tumor tissues, reflecting immune status in tumor microenvironments. 17, 28 Thus, the dynamic evaluation of systemic inflammatory factors may be helpful for estimating immune status changes during bevacizumab treatment. Moreover, although baseline systemic inflammatory factors have been found to predict the prognosis of NSCLC patients, 21-26 the dynamic changes may be more valuable in reflecting the real-time response to bevacizumab and be more accurate for predicting the patients' prognosis.

Our present study found that serum NLR, PLR, and SII in patients with CR/PR decreased consistently, but increased in patients with PD during the first 6 cycles of bevacizumab treatment; while there was an elevation of LMR in patients with CR/PR and reduction in patients with PD. This association between changes of systemic inflammatory factors and clinical response could at least be partly explained. More lymphocytes and less neutrophils and monocytes will migrate into local tissues and improve the immunological environment, which will lead to the prevention of tumor growth. In contrast, the increased neutrophil and monocyte and decreased lymphocytes presented with higher NLR, PLR, SII, and lower LMR, were found to demonstrate the tumor progression during bevacizumab treatment. More neutrophils and macrophages (derived from monocytic precursors) may move to tumor microenvironments and promote angiogenesis, not only by releasing proangiogenic factors VEGF, but also by triggering VEGF-independent angiogenesis, ultimately rendering the resistance to bevacizumab. 15, 29-32 Moreover, the resistance to bevacizumab will aggravate anaerobic condition in the tumor tissues attracting more neutrophils and macrophages, which will form the positive feedback loop in impairing the immune status and promoting the tumor progression.

Although the relationship between the systemic inflammatory factors and tumor infiltrating immune cells was not directly confirmed in our study, due to the difficulty in repeated acquirement of tumor tissues, previous studies have provided the evidence of successfully assessing immunological status in tumor microenvironments using the systemic inflammatory factors.^16^, ^17^ Nevertheless, we, for the first time, dynamically evaluated the kinetic changes of systemic inflammatory factors during bevacizumab treatment in NSCLC, and established the direct links between the changes of these factors and the tumor response. Activation of systemic immune status with the decline of serum NLR, PLR, and SII; and rise of serum LMR could indicate favorable response to bevacizumab, while the alteration to suppressive status indicate the resistance, which will serve as useful markers for the prediction of bevacizumab efficacy in an easy and non-invasive manner.

When the prognostic factors of these advanced NSCLC patients receiving bevacizumab treatment were analyzed, liver metastasis was found to independently predict both OS and PFS, consistent with previous studies.^33^ Due to the liver's important function in nutrient metabolism and detoxification, it is not surprising that NSCLC patients with liver metastasis showed a significantly poor prognosis compared to other distant metastasis. In the field of systemic inflammatory factors, only baseline LMR was found to predict a worse OS consistent with the literature; 34 however, other baseline factors lost their prediction significance in multivariate analysis of PFS and OS. Notably, dynamic changes of factors, such as increase of NLR and decrease of LMR were independently associated with OS of NSCLC patients with bevacizumab treatment, demonstrating that the dynamic changes of immune status played a more important role in the prognosis prediction. Dysregulation of immune system may cause the disruption of systemic homeostasis leading to the resistance to anti-tumor therapy and progression of tumor. Thus, systemic inflammatory factors were recommended to be dynamically considered when estimating the patients' prognosis, and were helpful in further guidance of individualized treatment for advanced NSCLC patients.

As indicators of immune response, NLR and PLR have been studied in the prediction of treatment response and outcomes in NSCLC patients receiving checkpoint inhibitor; the study reveals that elevated baseline NLR and PLR were associated with lower response rate and shorter OS and PFS when treated with nivolumab.^35^ Based on the preclinical findings, the immunosuppression induced by resistance of anti-angiogenesis might be settled by administration of immunotherapy. The survival benefit with respect to combination of atezolizumab and bevacizumab has been demonstrated in advanced NSCLC in IMpower150 clinical trial,^6^ suggesting the promising role of combination treatment in improving the patient outcomes; several other clinical trials are ongoing.^36^-^38^ Further studies are warranted to dynamically evaluate the predictive role of systemic inflammatory factors in the combination therapy. Nevertheless, we hope our results provide support for future studies examining the relationship between the changes of systemic immune status and the timepoint of combining bevacizumab and immunotherapy.

Our present study also has some limitations. First, the systemic inflammatory factors may be affected by other causes, such as bleeding and the use of steroids, which cannot be determined by a retrospective review. To eliminate confounding factors, patients with autoimmune diseases and active infections were excluded from our study. Second, high densities of CD4+ and CD8+ T cells in tumor tissues have been found to be favorable prognostic factors in NSCLC patients. However, we cannot analyze the specific subgroups of lymphocytes due to the retrospective nature. The role of specific subtypes of lymphocytes and inflammatory cytokines, such as interferon (IFN)-γ, IL-4 and IL-17, will be investigated in our further study. Lastly, the mechanisms underlying the influence of systemic inflammatory cells on the bevacizumab response still remain unclear; more work is urgently needed.

## Conclusion

Our results demonstrate that the activation of systemic immune status evaluated by the kinetic changes of serum inflammatory factors, was associated with good response to bevacizumab; while the alternation to suppressive status may indicate the resistance. In addition, dynamic changes of systemic inflammatory factors also had prognostic value in predicting outcomes of advanced NSCLC patients treated with bevacizumab. These non-invasive markers of systemic inflammatory factors are recommended to be assessed dynamically for obtaining more information to guide patient treatment in the future.

## Figures and Tables

**Table 1:** Characteristics of 161 study patients.

Parameter	No. of Patients (%)
Age, years	
≤57	85 (52.8%)
>57	76 (47.2%)
Sex	
Female	71 (44.1%)
Male	90 (55.9%)
Smoking History	
No	116 (72%)
Yes	45 (28%)
Anatomical location	
Peripheral	105 (65.2%)
Central	56 (34.8%)
EGFR	
Sensitive mutations	47 (29.2%)
Negative	79 (49.1%)
Resistant mutations	4 (2.5%)
Not available	31 (19.2%)
Bone metastasis	
No	100 (62.1%)
Yes	61 (37.9%)
Brain metastasis	
No	110 (68.3%)
Yes	51 (31.7%)
Liver metastasis	
No	135 (83.9%)
Yes	26 (16.1%)
Baseline NLR	
≤3.87	114 (70.8%)
>3.87	47 (29.2%)
Baseline PLR	
≤156	56 (34.8%)
>156	105 (65.2%)
Baseline SII	
≤824	98 (60.9%)
>824	63 (39.1%)
Baseline LMR	
≤2.37	55 (34.2%)
>2.37	106 (65.8%)

**Table 2:** Univariate and multivariate Cox models for PFS

Variables	Uni HR	95%CI	p-value	Multi HR	95%CI	p-value
Age	1.08	0.75-1.53	0.69			
Sex	1.19	0.83-1.71	0.34			
Smoking History	1.52	1.03-2.25	0.036			
Anatomical location	1.80	1.24-2.60	0.002	1.86	1.19-2.92	0.007
EGFR						
Negative	1.02	0.68-1.55	0.91			
Resistant mutations	2.69	0.64-11.2	0.18			
Bone metastasis	1.56	1.08-2.26	0.018			
Brain metastasis	1.06	0.87-1.29	0.57			
Liver metastasis	2.21	1.40-3.48	0.001	2.61	1.52-4.48	0.001
Baseline NLR	1.40	0.95-2.07	0.093			
Change of NLR	1.61	1.05-2.45	0.028			
Baseline PLR	0.83	0.58-1.21	0.33			
Change of PLR	1.02	0.67-1.55	0.94			
Baseline SII	1.38	0.96-1.98	0.08			
Change of SII	1.10	0.71-1.72	0.67			
Baseline LMR	0.67	0.46-0.97	0.035			
Change of LMR	0.59	0.38-0.91	0.015	0.62	0.4-0.96	0.033

**Table 3:** Univariate and multivariate Cox models for OS

Variables	Uni HR	95%CI	p-value	Multi HR	95%CI	p-value
Age	1.22	0.77-1.93	0.39			
Sex	1.33	0.84-2.11	0.23			
Smoking History	1.64	1.01-2.67	0.048			
Anatomical location	2.01	1.27-3.18	0.003			
EGFR						
Negative	1.25	0.72-2.14	0.43			
Resistant mutations	2.70	0.36-20	0.33			
Bone metastasis	1.80	1.14-2.86	0.012			
Brain metastasis	1.12	0.69-1.82	0.65			
Liver metastasis	2.27	1.31-3.92	0.003	2.47	1.23-4.99	0.01
Baseline NLR	1.50	0.92-2.44	0.10			
Change of NLR	2.29	1.31-4.00	0.004	2.36	1.25-4.44	0.008
Baseline PLR	0.71	0.25-1.13	0.15			
Change of PLR	1.18	0.67-2.06	0.57			
Baseline SII	1.42	0.90-2.26	0.14			
Change of SII	1.83	1.04-3.22	0.036			
Baseline LMR	0.52	0.33-0.84	0.007	0.40	0.17-0.94	0.036
Change of LMR	0.58	0.32-1.06	0.077	0.42	0.18-0.97	0.041

## Abbreviations

NSCLC: non-small cell lung cancer
NLR: neutrophil/lymphocyte
PLR: platelet/lymphocyte
SII: neutrophil×platelet/lymphocyte
LMR: lymphocyte/monocyte
LDH: lactate dehydrogenase
CRP: C-Reactive Protein
RECIST: Response Evaluation Criteria in Solid Tumors
ROC: receiver operating characteristics
PFS: progression free survival
OS: overall survival
CR: complete response
PR: partial response
SD: stable disease
PD: progressive disease
HR: hazard ratios
95%CI: 95% confidence intervals
